# An Assessment of Cell Culture Plate Surface Chemistry for *in Vitro* Studies of Tissue Engineering Scaffolds

**DOI:** 10.3390/jfb6041054

**Published:** 2015-11-26

**Authors:** Alexander Röder, Elena García-Gareta, Christina Theodoropoulos, Nikola Ristovski, Keith A. Blackwood, Maria A. Woodruff

**Affiliations:** 1 Institute of Health & Biomedical Innovation, Queensland University of Technology (QUT), Brisbane, Queensland, 4059, Australia; E-Mails: alexroeder89@gmail.com (A.R.); GarciaE@raft.ac.uk (E.G.-G.); c.theodoropoulos@qut.edu.au (C.T.); nikola.ristovski@hdr.qut.edu.au (N.R.); Raxxman@gmail.com (K.A.B.); 2 RAFT Institute of Plastic Surgery, Mount Vernon Hospital, Northwood HA6 2RN, UK

**Keywords:** tissue engineering, 3D scaffolds, cell attachment, poly-ε-caprolactone

## Abstract

The use of biopolymers as a three dimensional (3D) support structure for cell growth is a leading tissue engineering approach in regenerative medicine. Achieving consistent cell seeding and uniform cell distribution throughout 3D scaffold culture *in vitro* is an ongoing challenge. Traditionally, 3D scaffolds are cultured within tissue culture plates to enable reproducible cell seeding and ease of culture media change. In this study, we compared two different well-plates with different surface properties to assess whether seeding efficiencies and cell growth on 3D scaffolds were affected. Cell attachment and growth of murine calvarial osteoblast (MC3T3-E1) cells within a melt-electrospun poly-ε-caprolactone scaffold were assessed when cultured in either “low-adhesive” non-treated or corona discharged-treated well-plates. Increased cell adhesion was observed on the scaffold placed in the surface treated culture plates compared to the scaffold in the non-treated plates 24 h after seeding, although it was not significant. However, higher cell metabolic activity was observed on the bases of all well-plates than on the scaffold, except for day 21, well metabolic activity was higher in the scaffold contained in non-treated plate than the base. These results indicate that there is no advantage in using non-treated plates to improve initial cell seeding in 3D polymeric tissue engineering scaffolds, however non-treated plates may provide an improved metabolic environment for long-term studies.

## 1. Introduction

In recent years, the use of biodegradable biopolymers as a structural three-dimensional (3D) support material has become a prominent tissue engineering approach for repairing damaged cartilage or bone [1,2]. These biodegradable polymers can be either synthetic or derived from biological sources depending on the intended use [2]. One of the most widely used biopolymers, poly-ε-caprolactone (PCL), exhibits suitable properties for tissue engineering with good mechanical characteristics, established biocompatibility, and relatively long-term biodegradability [3]. The low melting temperature (~60 °C) of PCL also makes this material ideal for the fabrication of 3D porous scaffolds using an additive manufacturing or 3D printing technique. Cells, such as the bone-forming osteoblasts or bone marrow stromal cells, can be seeded and proliferated in PCL-scaffolds *in vitro* prior to transplantation [4,5,6,7].

While various studies focus on improving the structural, mechanical and fabrication aspects of PCL tissue engineering scaffolds [8,9], little attention has been given to quantifying cell attachment, proliferation and differentiation within these scaffolds in the period prior to transplantation. The homogeneity of the cultured tissue is dependent on how uniform the initial seeded cells are after being added to the scaffold [10]. As this knowledge is critical for designing successful tissue construct technologies, extensive *in vitro* cell culture studies are required to ascertain optimal seeding conditions.

Many static cell culture experiments are designed so that cells are seeded directly into a scaffold placed in a single well of a multi-well tissue culture plate. In many cases, the plates have been surface modified for the purpose of enhancing cell attachment. During the seeding process, some of the cells will attach to the scaffold fibers and some will, either through gravity or cell migration, attach or grow on the bottom surface of the tissue culture plate. As it is desirable to maximize cell-seeding efficiency on the scaffold for tissue engineering studies, various methods to achieve this have been developed. Some of these include reducing the cell suspension volume so that it is no greater than the volume of the scaffold and allowing 2–4 h for cell adherence before adding the final media volume [11,12]. Other approaches involve lining the base of the wells with 2% agarose or using tissue culture plastics with surface chemistry modifications for reduced cell attachment.

Various manufacturers use chemical and/or physical surface modifications in order to increase cell attachment to their plastic culture plates [13], often through techniques such as corona discharge [14]. This process typically uses plasma induced electric discharge to generate charge groups on the polystyrene surface of the culture plate to alter the surface chemistry from hydrophobic to hydrophilic. The objective is to enhance cell adherence, growth and differentiation in a 2D environment. For studies of cell growth within 3D scaffolds which are cultured within the well of tissue culture plates, enhanced cell adherence on the base of the tissue culture plate is undesirable and potentially leads to reduced cell seeding efficiency on the scaffold, and subsequent competition for nutrients as the cells proliferate. Given the importance of biocompatibility studies for *in vitro* assessment of the biomaterials and scaffolds, knowledge of the effects of tissue culture plate surface properties on cell seeding behavior in these contexts is vital.

In this study, we performed an assessment of cell seeding and growth on 3D melt-electrospun tissue engineering poly-ε-caprolactone (PCL) scaffolds cultured in both corona discharge-treated and “low-adhesive” tissue culture well-plates. This was achieved by seeding cells on melt-electrospun PCL scaffolds placed within tissue culture well-plates of each type, and performing periodic DNA quantification and cell metabolism analysis over four weeks. The results are intended to inform the choice of tissue culture well-plate materials for future *in vitro* studies of scaffold performance and optimization for tissue engineering applications. We hypothesize that low adhesive plates would enable a greater cell attachment to the PCL scaffolds owing to cells preferentially attaching to PCL compared to treated tissue culture plates which cells might attach to in preference, thus, leading to lower seeding density on PCL scaffolds in treated tissue plates.

## 2. Results and Discussion

### 2.1. Scaffold Fabrication and Biocompatibility

**Figure 1:**
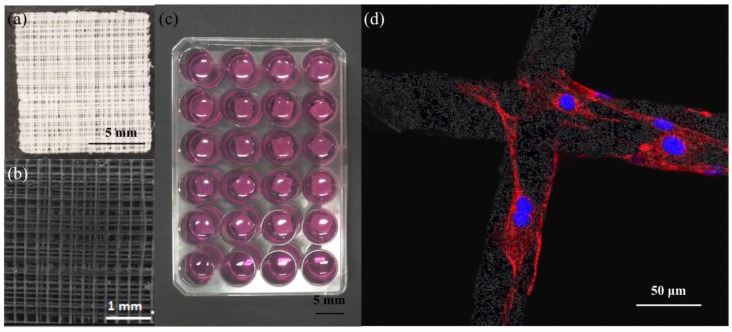
Assessment of melt electrospun poly-ε-caprolactone (PCL)-scaffolds. (**a**) Photograph of 10 mm × 10 mm square of melt-electrospun PCL-scaffold; (**b**) High resolution micrograph demonstrating ordered 0–90° laydown pattern achieved with melt-electrospinning; (**c**) Representative image of the cell culture set-up of MC3T3-E1 cell-seeded scaffolds in multi-well plates; (**d**) Representative confocal laser scanning micrograph of MC3T3-E1 cells adhering to PCL-scaffold. Red: Alexa-Fluor 568 Phalloidin conjugates (actin), blue: DAPI (nuclei). Scale bars as shown.

Poly-ε-caprolactone was successfully melt-electrospun into 400 mm × 400mm fibrous sheets (Figure 1a). High resolution analysis by scanning electron microscopy demonstrated the ordered 0–90 °C laydown pattern (Figure 1b). To evaluate the biocompatibility of the scaffold, DAPI/Phalloidin staining of the cells was analyzed. At 24 h, single cells were observed adhering to the fibres (see Figure 1d), and by day 7, cells were stretched out along the fibres and starting to bridge the gap between two intersecting fibres. At day 14, cells had formed continuous sheets closing the scaffold pores (data not shown). There were no differences in cell morphology for those on scaffolds grown in both treated and non-treated well-plates for the duration of the study.

### 2.2. Comparison of Different Commercial Tissue Culture Surfaces on Scaffold Cell Seeding and Proliferation

**Figure 2:**
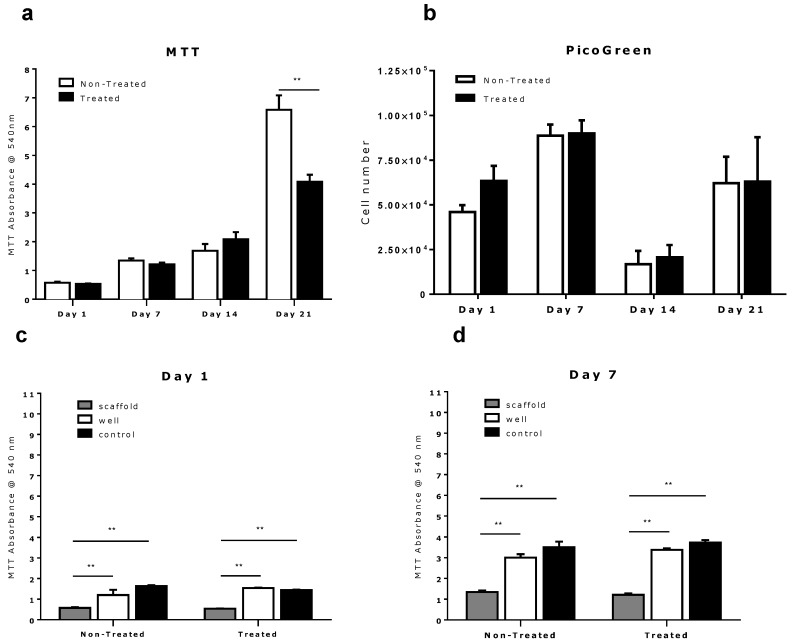

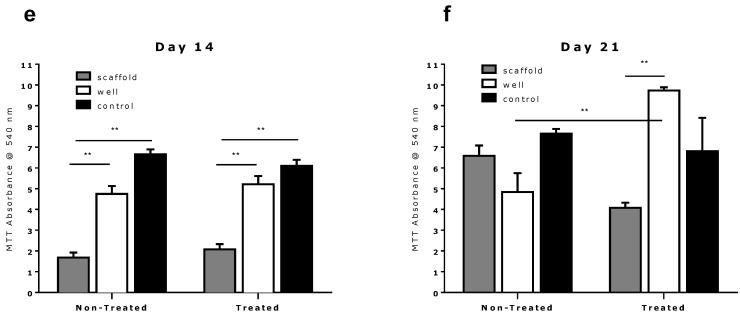
Quantitative analysis of cell growth and proliferation on melt-electrospun scaffolds when cultured in treated *versus* non-treated tissue culture multi-well plates. (**a**) A higher level of metabolic activity as assessed by MTT was detected on day 21; (**b**) No difference in total DNA content is observed via the PicoGreen^®^ assay; (**c**,**d**) MTT assay shows no significant difference in scaffold-based cell proliferation between multi-well plates for day 1, 7 and 14, but an increased cell metabolic activity in scaffolds kept in the non-treated multi-well plate at day 21 (**a**); (**c**–**e**) MTT assay shows a significantly higher cell metabolic activity of bottom-based cells compared to scaffold-based cells at day 1, 7 and 14. Significance was calculated using unpaired 2-tailed student’s *t*-test. Results are presented as mean with SEM. ** indicates *p* < 0.005.

To evaluate the effect of tissue culture well-plate surface chemistry on cell seeding and growth on 3D tissue engineering scaffolds which we placed into the well *in vitro*, DNA quantification and cell metabolism were analyzed over a three-week period (days 1, 7, 14 and 21). Figure 2a,b indicate that MC3T3-E1 cells attached and proliferated on PCL-scaffolds cultured in both the treated and untreated tissue culture well-plates. We also observed that in the 24 h following seeding, a higher amount of DNA was recovered from the scaffold cultured in the treated well-plate than from the scaffold cultured in the non-treated well-plate. This difference, however, became less apparent in following measurements at days 7, 14 and 21 (Figure 2b). A significant decrease in DNA concentration was also apparent on all scaffolds in measurements taken after day 7. This is consistent with other studies and has been previously attributed to insufficient DNA extraction from cells “entrapped” in polymer matrix of the 3D PCL-scaffold [15,16,17,18]. Due to the difficulty in consistently extracting DNA from deep within the 3D environment for the PicoGreen^®^ assay, we used the MTT metabolic activity assay for cell number estimation. This was due to the easier elution of the tetrazolium dye from deep within the scaffolds. Although this assay does not directly measure cell number, but it can measure cell metabolism thus, be used as a comparative measure of cellular activity across the scaffolds under similar culture conditions. This assumption enables the MTT results to provide an indirect measure of cell proliferation.

The MTT assay revealed no significant difference in viable cell metabolism for scaffolds cultured in either the non-treated or the treated multi-well plate up to day 14 (Figure 2a). However, at day 21 we observed a significantly higher metabolism from the PCL scaffolds cultured in non-treated well-plates. Further analysis revealed this increased scaffold cell activity correlates with a relative reduction in the cell activity on the corresponding well-plate as shown in Figure 2f). This behavior was not apparent on the scaffolds in the surface treated well-plate, with higher cell activity on the well-plate than on the scaffold. Aside from this result, a higher cell metabolic activity was generally observed on the base of tissue culture well-plates than on the scaffolds throughout the entire study (Figure 2c–f). The control group of culture plates containing no scaffolds also showed higher cell activity than all other groups in nearly all cases.

The higher cell activity seen on both types of well-plates indicate that the cells do in fact preferentially attach to the base of culture plates following seeding (see Figure 2c). As these well-plates have very different surface chemistry, this suggests that the principle mechanism behind the higher cell attachment and activity on the well-plates is not chemical, but potentially due to gravity.

The results also suggest that there is no evidence of statistically different cell activity on the scaffolds cultured in either the treated or non-treated cell culture well-plates (*p* < 0.05) for days 1, 7 and 14.

On day 21, however, we observed a greater cell activity on the scaffold cultured in the non-treated well-plate than on the base of the well-plate in contrast to observations made at previous time points. However, this greater cell activity was not observed in the corresponding surface treated well-plates. In surface treated well-plates significantly greater cell activity was measured from the base of the well-plate than on the scaffold, exceeding the cell activity measured on the corresponding control treated culture plate containing no-scaffold (see Figure 2f). This may suggest that the corona discharge surface treatment of tissue culture plates produces a more preferable metabolic environment for cells compared to PCL following longer duration studies. One possible interpretation is that cells eventually out compete their scaffold-based counterparts for nutrients (Figure 2f), leading to a reduction in cell proliferation within the scaffold in treated tissue culture plate. This is the opposite of the behavior seen in the non-treated well-plates where it appears that the cell activity is higher in the PCL scaffold.

Generally, a 27% greater cell metabolic activity on average was seen in the PCL scaffolds and corresponding tissue culture well-plates than on the control group of well-plates containing no PCL scaffolds. This indicates that the larger surface area provided by the 3D scaffolds provides a preferable environment for cell expansion than the 2D cell culture well-plates.

## 3. Experimental Section

### 3.1. Melt-Electrospun Scaffolds

Poly-ε-caprolactone (PCL, Capa 6430^®^, Perstorp UK Limited) scaffolds were fabricated as described previously using melt electrospinning [9]. Scaffold sheets (400 mm × 400 mm × 2 mm) were fabricated to have a stacked (50 layers) 90° cross-hatched microarchitecture, with a fiber thickness of 50 µm, and spacing of 200 µm (Figure 1a,b). Prior to use, scaffolds were sectioned with a sharp blade into 10 × 10 mm identical squares (Figure 1a). Fiber surface modification was performed to reduce hydrophobicity of the PCL by immersing the scaffolds in 5 M NaOH for 90 min at room temperature [19]. The scaffolds were then rinsed several times in MilliQ water until the rinsed water pH reduced to 7.0, and were left to dry overnight at 40 °C in a desiccator. Prior to cell seeding, the PCL scaffolds were sterilized with ultraviolet light for 2 h on each surface.

To evaluate the PCL-electrospun scaffold architecture, light and scanning electron microscopy (see Figure 1a,b respectively) were performed. The scaffolds were sputtered coated with gold using the Leica EM SCD005 sputter coater prior to imaging at 10 kV using a FEI Quanta 200 scanning electron microscope (FEI Company, Eindhoven, Netherlands). 

### 3.2. Multi-Well Plates

To determine the effect of different surface characteristics of tissue culture well plates on cell seeding efficiency and subsequent tissue formation in 3D PCL scaffolds, two commonly used multi-well plates were tested: Thermo Scientific™ Nunc™ Non-Treated 24-multi-well plates (cat #144530) and Corning Costar^®^ TC-Treated 24-multi-well plates (cat #3524). The PCL scaffolds were equally divided into these 2 groups, and placed into the individual wells of each respective plate (Figure 1c).

### 3.3. Cell Culture

Murine calvarial osteoblast cells MC3T3-E1 (100,000 in 140 µL culture medium) were seeded directly onto each PCL scaffold and allowed to adhere for 90 min at 37 °C with 5% CO_2_ prior to filling the well with an additional 2 mL of proliferation culture media (Minimum Essential Medium-Alpha containing l-Glutamine, Ribonucleotides and Deoxyribonucleosides (MEM-α, Life Technologies, Grand Island, NY, USA) supplemented with 10% (v/v) fetal bovine serum (Lonza Australia, Mount Waverley, Australia) and 1% (v/v) penicillin/streptomycin (10000 U/mL) (Life Technologies, Grand Island, NY, USA)). Additionally, 100,000 MC3TC-E1 cells in 140 µL culture medium were seeded into wells of each tissue culture plate type, in the absence of a scaffold, to serve as a control. These cells were allowed to adhere for 90 min at 37 °C with 5% CO_2_ prior to filling the well with an additional 2 mL of proliferation culture media. Cultures were incubated at 37 °C with 5% CO_2_ until respective time-points were reached, and medium was replaced every other day.

### 3.4. Cell Morphology and Attachment

At selected time points (days 1, 7, 14 and 21) (*n* = 3) cell attachment on the PCL scaffold was assessed qualitatively by staining with 5 µg/mL 4,6-diamino-2-phenylindole (DAPI, Life Technologies, NY, USA) and 0.8 U/mL Alexa Fluor 568 Phalloidin (Life Technologies, Grand Island, NY, USA) as described previously [20]. Imaging was undertaken using the Leica TCS SP5 scanning laser confocal microscope (Leica Microsystems, Mannheim, Germany). 

### 3.5. Quantitative Analysis

#### 3.5.1. Cell Metabolic Activity Assay-MTT

To assess the metabolic activity of cells when cultured on scaffolds placed in each tissue culture multi-well plates, (3-(4,5-Dimethylthiazol-2-yl)-2,5-diphenyltetrazolium bromide), Sigma-Aldrich, Oakville, ON, Canada) assay was used. In brief, at days 1, 7, 14 and 21 (*n* = 6), cell seeded PCL-scaffolds were transferred into a fresh multi-well plate at respective time points. 500 µL of unused cell proliferation medium supplemented with 0.25 mg/mL MTT solution was immediately added to: (1) the scaffolds (*n* = 3); (2) empty multi-well plate after scaffold removal (*n* = 3); and (3) control well seeded with cells only (*n* = 3). The MTT solution was incubated at 37 °C with 5% CO_2_ for 4 h. Media was removed and 500 µL (day 1), 1000 µL (day 7) or 2000 µL (day 14 and 21) dimethyl sulfoxide (DMSO, Merck Millipore, Darmstadt, Germany) was added to each sample. These dilution volumes were deliberately chosen based on previous observations [20] to keep the OD readings within the accuracy of the plate reader; lower than 1 (10% light). The plates were then covered with aluminum foil and placed on an orbital shaker for 10 min prior to 100 µL of DMSO elutant transferred from each well and into a fresh 96-well multi-well plate and absorption measured at 540 nm using a Benchmark Plus™ Multi-well plate Spectrophotometer (Bio-Rad Laboratories, Hercules, California, USA).

#### 3.5.2. DNA Content—Picogreen

At each time-point (days 1, 7, 14, 21; *n* = 6), samples (both cell seeded scaffold, as well as cells grown in the base of a well-plate), were washed with PBS twice, rinsed with 0.2% Triton™ X-100 (Sigma) in 1× TE buffer and frozen, and thawed twice before analysis. The assay to determine the amount of DNA per sample was then performed using PicoGreen^®^ dsDNA quantification kit (Quant-iT™ PicoGreen^®^ dsDNA assay kit, Life Technologies, Grand Island, NY, USA) following the manufacturer’s instructions. Briefly, samples were diluted 1:9 (days 1 and 7) or 1:4 (days 14 and 21) (lysate: ×1 TE Buffer) in fresh black 96-well plates and analyzed in triplicate. Fluorescence readings were recorded using the POLARstar Optima multi-well plate reader (BMG Labtech, Offenburg, Germany) with an excitation wavelength of 485 nm and an emission filter of 520 nm. The amount of cells attached per scaffold and per well was calculated based on dsDNA content per cell, with one cell assumed to contain 7.7 pg DNA [21].

### 3.6. Statistics

Significance was calculated using unpaired 2-tailed student’s T-test using GraphPad Prism software Version 5.03 for Windows. Mean with SEM are presented in all figures. *p* < 0.05 were considered significant.

## 4. Conclusions

In conclusion, we demonstrated no significant difference in cell seeding efficiency and proliferation on melt-electrospun PCL tissue engineering scaffolds, cultured in either surface or non-surface treated tissue culture well-plates, over a two week period. This result was surprising given that the surface treated well-plates are specifically modified to provide an improved environment for cells, and therefore would be expected to provide a less optimal environment for PCL scaffolds which were contained in the treated culture plates. We did, however, observe differences in cell metabolic activity on PCL scaffolds cultured in both types of well-plates at day 21, with cells appearing to prefer the 3D scaffold environment over the base of the well-plate in the case of the non-treated surface groups, and prefer the base of the well-plate over the scaffold in the case of surface treated well-plate groups. These results suggest that the choice of tissue culture well-plate surface chemistry does not significantly affect cell seeding efficiency and proliferation in the early stages of *in vitro* tissue engineering scaffold studies. However, non-treated well-plates may provide an improved metabolic environment than surface treated well-plates for longer duration studies.
